# Simplified evaluation of energetic complementarity based on monthly average data

**DOI:** 10.1016/j.mex.2019.05.019

**Published:** 2019-05-18

**Authors:** Alexandre Beluco, Alfonso Risso, Fausto A. Canales

**Affiliations:** a Instituto de Pesquisas Hidráulicas (IPH), Universidade Federal do Rio Grande do Sul (UFRGS), Porto Alegre, Rio Grande do Sul, Brazil; b Centro Estadual de Pesquisas em Sensoriamento Remoto e Meteorologia, Universidade Federal do Rio Grande do Sul (UFRGS), Porto Alegre, Rio Grande do Sul, Brazil; c Department of Civil and Environmental, Universidad de la Costa, Barranquilla, Atlantico, Colombia

**Keywords:** Method for determination of energetic complementarity from monthly average data, renewable energy, energetic complementarity, hybrid energy systems

## Abstract

Energetic complementarity is a subject that has been holding more and more attention from researchers in recent years, being a concept that can be applied both in energy planning stages and in phases of operation of energy systems based on renewable energy resources. The complementarity between two renewable sources of energy has three components: time-complementarity, energy-complementarity and amplitude-complementarity, and can be determined between raw energy availabilities or between energy generated by power plants. Complementarity can be evaluated between two renewable resources in the same place or between two renewable resources in different places and these two types can be denominated respectively as temporal and spatial complementarity. This method allows simplified evaluation of the energy complementarity between two renewable resources by comparing basic parameters obtained from series of monthly average values that characterize these resources. Finally, an application example clarifies the application of the method.

• The method allows a quick and visual but expeditious evaluation of energetic complementarity.
• This method provides a reference value for the application of more complex methods for evaluation of complementarity.
• Monthly average data allows the comparison of renewable resources with different characteristics of intermittency and variability;

## Graphical abstract

**Figure:**
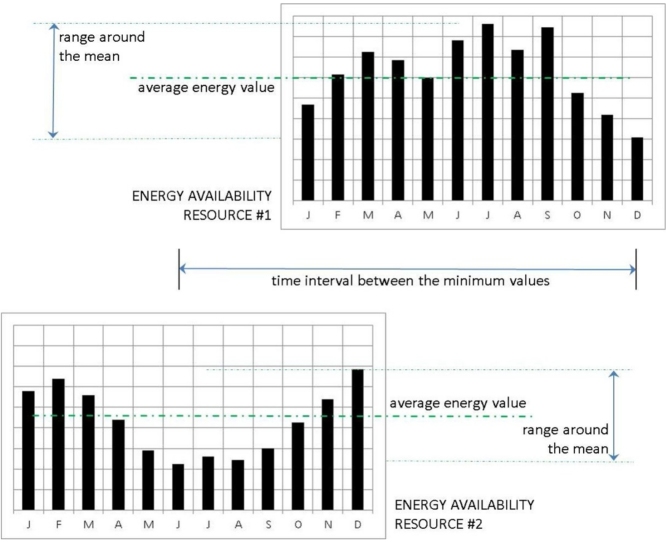

**Specifications Table**

**Table:** 

Subject Area:	*Energy*
More specific subject area:	*Renewable Energy – Hybrid Energy Systems*
Method name:	*Method for determination of energetic complementarity from monthly average data*
Name and reference of original method:	*A dimensionless index evaluating the time complementarity between solar and hydraulic energies. Beluco et al.* [1]*. Renewable Energy (2008), v.33, n.10, p.2157-2165.*
Resource availability:	*N/A*

## Method details

### Background

As discussed by Beluco et al. [1] in his article published in 2008, energetic (or energy) complementarity can be understood as the ability of two (or more) renewable resources to present complementary energy availabilities. This complementarity can also be identified in the energy supplied by power plants. Energetic complementarity can be identified between different renewable resources in the same place, or between renewable resources in different locations. Complementarity at the same site may be referred to as temporal complementarity, while complementarity identified between different sites may be referred to as spatial complementarity. This article by Beluco et al. [1] only discusses the concept of complementarity and proposes a dimensionless index that allows quantifying the complementarity between two energy resources, without explicitly proposing a method for assessing complementarity. Over the years, the first author noticed some specific and recurring difficulties among researchers interested in exploring the theme of complementarity and who expressed doubts in the qualification and quantification of complementarity. The complementarity between energy resources at different sites requires techniques for graphically expressing the results, as discussed and proposed by Risso et al. [2] recently, but the quantification of spatial complementarity is done with a protocol similar to quantification for temporal complementarity. A more precise evaluation of complementarity may require more complex mathematical methods, such as that proposed by Borba and Brito [3] and that allow to work with more than two energetic resources, but an understanding of what complementarity is obligatorily includes the evaluation of at least some series of average monthly data, for a better visual understanding of what actually happens. This method article presents some simple tricks developed by the authors over the years in which they have been exploring the theme of complementarity. Throughout this article, as the subject is the quantification of complementarity, the expressions 'complementarity' and 'complementarity index' will be used interchangeably as synonyms.

### Method

The simplified method for determining energetic complementarity from the average monthly data consists of the following steps:

1 Characterize the energetic complementarity to be achieved.

Note. This step can be accomplished by answering the following questions: Should complete complementarity or some of its components be determined? In case some components are sufficient, time-complementarity, energy-complementarity or amplitude-complementarity must be determined? Will raw data or final data (or other data qualification) be used?

2 Selecting the data series will be used to characterize the two energy resources whose complementarity will be evaluated.

Note. The lack of long series of field data hampers any analysis and the necessary selection of series of the same length for the two energy resources can overestimate extreme events. The decision on data related to the final energy supplied by plants favors a less speculative complementarity assessment.

3 Determine the month in which the minimum value of availability of the first energy resource occurs, calling this variable as m_1_. This variable should indicate the month of the year and should be a number between 1 and 12.
4 Likewise, determine the month in which the minimum value of availability of the second energy resource occurs, calling this variable as m_2_. This variable should indicate the month of the year and should be a number between 1 and 12.
5 Determine time-complementarity component, κ_t_, comparing m_1_ e m_2_ and choosing the smallest value between the results of the following two equations:

$${\text{κ}}_{\text{t}}=\frac{\left|{\text{m}}_{1}-{\text{m}}_{2}\right|}{6}\;{\text{κ}}_{\text{t}}=\frac{\left|{\text{m}}_{2}-{\text{m}}_{1}+12\right|}{6}$$

Notes. This equation is a monthly scale adaptation of the equation presented in ref. [1]. The result will be equal to 0 if the months for the minimum values coincide and therefore no complementarity occurs. The result will be 1 if the months have a 6 month gap between them, setting up full complementarity. The intermediate values obviously present linear variation.

The adoption of the lower of the two results is suggested because the complementarity in time should be evaluated based on the smaller distance between the minimum values of availability of the two energetic resources under study.

6 Determine the average value of availability of the first energy resource, denominating this variable as e_1_.
7 Likewise, determine the average value of availability of the second energy resource, denominating this variable as e_2_.
8 Determine energy-complementarity component, κ_e_, with the following equation:

$${\text{κ}}_{\text{e}}=1-\left|\frac{{\text{e}}_{1}-{\text{e}}_{2}}{{\text{e}}_{1}+{\text{e}}_{2}}\right|$$

Note. The result will be equal to 1 if the two mean values are equal and will be 0 at the limit if one of the two values is much larger than the other. The intermediate values present a non-linear relation between them.

9 Determine the amplitude of variation around the average value of availability of the first energy resource, denominating this variable as d_1_. This value is just the difference between the maximum and minimum availability of the first resource.
10 Likewise, determine the amplitude of variation around the average value of availability of the second energy resource, also only the difference between the maximum and minimum availability of the second resource, denominating this variable as d_2_.
11 Determine energy-complementarity component, κ_a_, with the following equations:

δ1=1-d1e1 δ2=1-d2e2 κa= 1-δ1-δ21-δ22 se δ1≤δ21-δ221-δ22+δ1-δ22 se δ1≥δ2

Note. The result will be equal to 1 if the two differences d_1_ and d_2_ are equal and will equal 0 at the limit if one of them is much larger than the other. The intermediate values present a non-linear relation between them.

12 Determine the final value of complementarity, κ, by multiplying the values obtained for the partial indexes κ_t_, κ_e_ and κ_a_, determined respectively in steps 5, 6 and 7 above.

Note. The final value for the complementarity index will be a value between 0 and 1, where the extreme values 0 and 1 correspond respectively to zero complementarity and full complementarity. If any of the three components assumes a very low value, the complementarity assessment can be pushed too low. For this reason, an analysis considering both the time component and the final complementarity was suggested.

### Application example

**Fig. 1:**
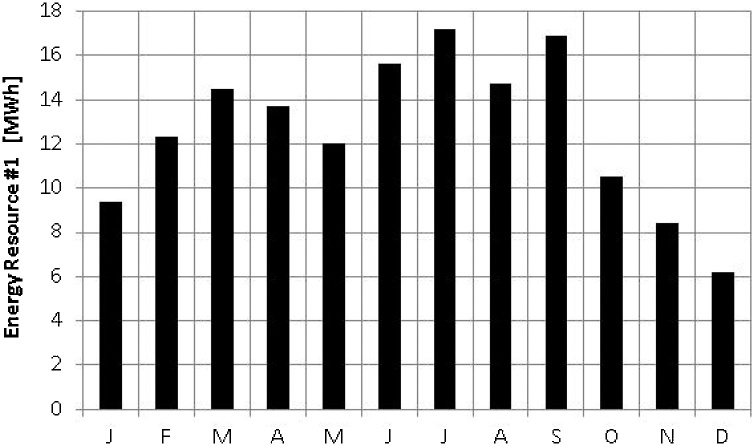
Monthly average energy availability for renewable resource #1.

**Fig. 2:**
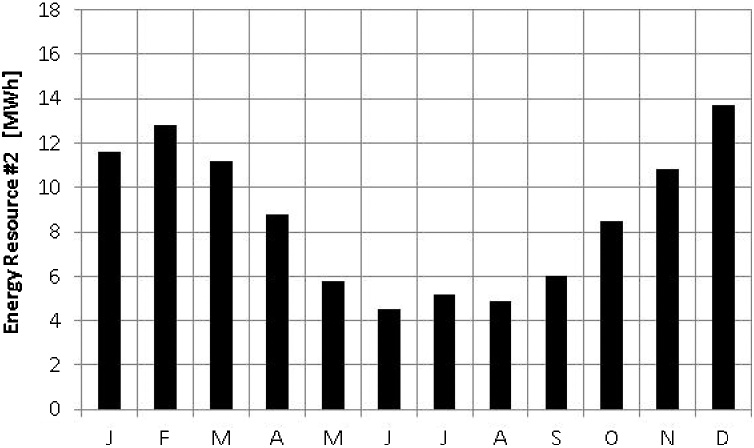
Monthly average energy availability for renewable resource #2.

As an example of application of the method in this paper, Fig. 1, Fig. 2 show monthly average energy availabilities from two renewable sources and the complementarity between these two energy resources will be determined. Following the steps outlined in the previous section, the first step indicates the qualification of energetic complementarity to be determined and the second the selection of data to be used in the analysis. In this example, the figures present final values of energy, obtained with power plants in operation and, thus, complementarity will be obtained for the energy to be supplied directly to the consumers.

The third and fourth steps of the previous section indicate the determination of the months in which the resources present their minimum values of energy availability. Thus, the renewable energy resource in Fig. 1 presents a minimum value in December, while the renewable energy resource in Fig. 2 presents a minimum in June. The variable m_1_ will then be equal to 12, while m_2_ will equal 6. The fifth step indicates the equation for the determination of κ_t_ and the result will be equal to 1.00, (since m_1_ minus m_2_ will equal 6, then divided by 6) indicating full time-complementarity.

In this example, the time lag between the months in which the minimum availability occur is 6 months. If, by chance, the minimum value of the second resource occurred in the month of April rather than June, the two values would be 1.33 (which is inconsistent with the proposal that the complementarity index vary between 0 and 1) and 0.67 and the final value would therefore be 0.67.

The sixth and seventh steps of the above method indicate the determination of the average values of available energy. The resource in Fig. 1 shows an average of 12.6 MW h, while the resource in Fig. 2 shows an average of 8.7 MW h. Thus, the variable e_1_ will equal 12.6 MW h, while e_2_ will equal 8.7 MW h. The eighth step indicates the equation for the determination of κ_e_ and the result will be equal to 0.82, indicating a relatively high complementarity.

The ninth and tenth steps, in turn, indicate the determination of the amplitudes of variation of the two energy resources. The resource in Fig. 1 shows an amplitude of variation of 11.0 MW h, while the resource in Fig. 2 shows an amplitude of variation of 9.2 MW h. In Fig. 1, the minimum occurs in December and the maximum in July and, in Fig. 2, the minimum occurs in June and the maximum in December. Thus, d_1_ will equal 11.0 MW h, while d_2_ will equal 9.2 MW h. The eleventh step guides how to arrive at the value of κ_a_ from d_1_ and d_2_ and the result is 0.96, also indicating a fairly high partial complementarity.

Finally, the twelfth step of the method described above indicates the determination of the total complementarity index from the product of the three components respectively determined at the fifth, eighth and eleventh steps. The result indicates a final value for complementarity equal to 0.79, indicating a relatively high value for complementarity.
